# Annexin A1 Is Involved in the Antitumor Effects of 5-Azacytidine in Human Oral Squamous Carcinoma Cells

**DOI:** 10.3390/cancers17071058

**Published:** 2025-03-21

**Authors:** Nunzia Novizio, Raffaella Belvedere, Mariangela Palazzo, Silvia Varricchio, Francesco Merolla, Stefania Staibano, Gennaro Ilardi, Antonello Petrella

**Affiliations:** 1 Department of Pharmacy, University of Salerno, Via Giovanni Paolo II 132, 84084 Fisciano, Italy; nnovizio@unisa.it (N.N.); rbelvedere@unisa.it (R.B.); marpalazzo@unisa.it (M.P.); 2 Pathology Section, Department of Advanced Biomedical Sciences, University of Naples “Federico II”, Via Sergio Pansini 5, 80131 Naples, Italy; silvia.varricchio@unina.it (S.V.); staibano@unina.it (S.S.); gennaro.ilardi@unina.it (G.I.); 3 Department of Medicine and Health Sciences “V. Tiberio”, University of Molise, Via F. De Sanctis, 86100 Campobasso, Italy; francesco.merolla@unimol.it

**Keywords:** 5-azacytidine, annexin A1, cell motility, mesenchymal-to-epithelial transition, oral squamous cell carcinoma

## Abstract

**Background:** the treatment of squamous cell carcinomas of the oral cavity (OSCCs) is limited by the lack of reliable diagnostic/prognostic, and predictive markers, as well as by intrinsic tumor cell heterogeneity. 5-azacytidine (5-AZA) offers opportunities for cancer cell reprogramming to develop new target-specific treatments. The protein annexin A1 (ANXA1) is downregulated in head and neck squamous cell carcinoma (HNSCC), correlated with pathological differentiation grade. **Objectives:** this work aimed to further investigate the role of ANXA1 in OSCC progression based on 5-AZA activity. **Methods:** we used CAL27 and CAL33 cell lines, which differ in drug sensitivity and differentiation status. **Results:** CAL27 showed a higher expression of the stemness markers compared to CAL33 cells, but this positivity was lost after treatment with 5-AZA. This drug also decreased CAL27 cell motility, promoting a less aggressive phenotype. Moreover, 5-AZA increased ANXA1 expression only in CAL27. After siRNA-mediated downmodulation, we witnessed a significant rise in cell motility and the inversion of E-/N-cadherin expression, which was reverted again by 5-AZA. To investigate the role of exogenous ANXA1 derived from the tumor microenvironment, we treated CAL27 with Ac2-26, an ANXA1 mimetic peptide. Interestingly, we found that this peptide alone showed impacts similar to 5-AZA in reversing the aggressive phenotype. All these effects were not evidenced in CAL33 cells. Finally, to prove the loop of the exogenous protein, we detected increased expression of its receptors, formyl peptide receptors (FPRs), and their activation, leading to oncosuppressor effects. **Conclusions:** we propose that ANXA1 mediates the effects of 5-AZA only in poorly differentiated stemlike CAL27 cell lines. This suggests the relevance of ANXA1 as a diagnostic/prognostic biomarker in OSCCs, paving the way for personalized therapies to overcome treatment difficulties.

## Simple Summary

In order to improve the therapeutic treatment of oral squamous cell carcinomas (OSCCs), we proposed to use 5-AZA as an adjuvant drug in the management of this cancer, which is often associated with resistance and relapse. These factors are also associated with intrinsic tumor cell heterogeneity. Here, for the first time, we have characterized two human cell lines, CAL27 and CAL33, which are used as cell models in OSCCs, by studying the stemness marker staining and their differential responses to 5-AZA. We also investigated how this drug could influence OSCC ANXA1 expression and how this regulation could significantly affect the cell phenotype.

## 1. Introduction

Squamous cell carcinoma of the head and neck (HNSCC) is the sixth most common cancer worldwide [1]. Squamous cell carcinomas of the oral cavity (OSCCs), the most frequent form of HNSCC [2], affect every subsite of the oral cavity, mainly the tongue and the floor of the mouth. Despite recent advances in the investigation and therapeutic approaches, OSCC survival has only slightly improved, and mortality remains high [3,4]. Moreover, OSCCs are characterized by genetic and molecular heterogeneity and diversity, which result in different cell subpopulations with diverse functional phenotypic forms within the same tumor and in tumors with the same localization. These subpopulations include tissue-specific cancer stem/progenitor cells, post-mitotic cancer cells at different stages of maturation, and de-differentiated cancer cells. All of these have a significant impact on the biological behavior, treatment response, and the generally poor prognosis of this carcinoma [5]. In OSCCs, the first line of treatment is surgical resection, often associated with radiotherapy and/or chemotherapy in cases of advanced disease, or risk of recurrence or resistance [6,7]. Some strategies have been applied in patients to prevent chemotherapy resistance, including treatment with 5-azacytidine (5-AZA) [8], a DNA demethylating agent [9,10]. Indeed, in several preclinical studies, it has been shown that treatment with DNA demethylating agents may help or restore chemotherapy sensitivity, correlated with the expression of specific proteins [11,12,13]. The biological action of 5-AZA reflects the polyvalent inhibitory mechanism of this drug: firstly, by inhibiting RNA and DNA methylation, by depressing maturation of ribosomal RNA, and, in the last instance, by interacting with pyrimidine synthesis de novo and incorporating itself into RNA and DNA with a cytotoxic effect [14].

For these reasons, 5-AZA is involved in cell growth and differentiation in different models [9,10] and is associated with increased transcriptional activity of tumor suppressor candidate genes [8,13]. HNSCC, and also OSCCs, have resulted as sensitive to 5-AZA in clinical trial and experimental data [15,16,17].

Currently, prognostic and predictive factors regarding the biological aggressiveness of OSCCs are exiguous [18]. Thus, it is crucial to find proteins that can be considered as reliable molecular biomarkers for OSCCs in diagnostic/prognostic purposes and as new potential therapeutic targets. Several clinical studies have suggested annexin A1 (ANXA1) as a prognostic biomarker of OSCCs [19,20], mainly reporting that its expression, in OSCC patients, is related to the pathological differentiation grade. Furthermore, Wan et al. [21], for the first time, studied the role of this protein in OSCC cell proliferation and in vitro invasion, two mechanisms involved in tumor progression. In detail, they demonstrated that forced overexpression of this protein in OSCC cell lines, such as CAL27 and Tca8113, significantly reduced proliferation and invasiveness with increased expression of E-cadherin and repression of vimentin and N-cadherin levels. It is well known that ANXA1, belonging to the annexin family, participates in several pathophysiological mechanisms [22], also triggered by its interaction with formyl-peptide receptors (FPRs) [23]. Since its discovery as an anti-inflammatory protein, the involvement and therapeutic potential of ANXA1 in various diseases have been proven, including atherosclerosis, stroke, neurodegenerative disorders, pulmonary and renal fibrosis, and rheumatoid arthritis [24].

Regarding cancer, a growing number of studies support that ANXA1 is differentially expressed in many kinds of tumors, and it is considered as a tissue-specific oncogene or oncosuppressor [25,26]. In HNSCC, ANXA1 appears to be downmodulated, acting as an oncosuppressor [27].

Given the ability of 5-AZA to induce the transcription of tumor suppressor genes and the possible role of ANXA1 as a marker of OSCCs, in our study, we assessed the effect of this drug, used as an adjuvant treatment in OSCCs, focusing on ANXA1 expression. Furthermore, the aim of the present study was to investigate ANXA1’s biological activity on two different OSCC HPV- cancer cells from the tongue, also in correlation with its overexpression induced by 5-AZA, in terms of cell motility and phenotypic changes.

## 2. Materials and Methods

### 2.1. Cell Culture

CAL27 cells are immortalized epithelial cells isolated from tissue taken prior to treatment from a patient with a lesion in the middle of the tongue in 1982. This cell line was purchased from the American Type Culture Collection (ATCC) (CRL-2095, ATCC; Manassas, VA, USA). CAL33 cells are immortalized epithelial cells established from the surgically removed fragment of a tongue lesion from a patient with moderately differentiated squamous cell carcinoma of the tongue in 1983 (prior to therapy). This cell line was obtained from DSMZ (ACC447, Leibniz Institute DSMZ; Braunschweig, Germany). These cell lines have already been characterized for their morphology, ultrastructure, karyotype, immunological properties, and response to various cytotoxic drugs used for cancer treatment. In detail, both cell lines have been described as having a polygonal form. While CAL27 cells present a highly granular cytoplasm, CAL33 cells show nucleus with one, two, or three visible nucleoli and retain a more uniform size. Cytogenetic examination displayed that both lines were malignant and were tumorigenic after inoculation into nude mice. Overall, CAL33 cells were defined as more differentiated than CAL27 cells, and the latter were found to be more resistant to drug treatments than the CAL33 counterpart [28].

Both cell lines were grown in Dulbecco’s modified Eagle medium (DMEM; Euroclone s.p.a.; Milan, Italy) supplemented with 10% (*v*/*v*) fetal bovine serum (FBS; Sigma-Aldrich; St. Louis, MO, USA) at 37 °C in a 5% CO_2_ (*v*/*v*) humidified air atmosphere.

### 2.2. Chemicals and Reagents

5-Aza-2′-deoxycytidine (5-AZA) was purchased from Sigma-Aldrich (Sigma-Aldrich; St. Louis, MO, USA). The powder was dissolved in dimethyl sulfoxide (DMSO; Sigma-Aldrich, St. Louis, MO, USA) in order to obtain the 50 mM concentration and used at 25 μM, 100 μM and 200 μM, as reported in [15].

Mitomycin C was obtained from Sigma-Aldrich (Sigma-Aldrich; St. Louis, MO, USA), dissolved in a solution with 10% MeOH in bidistilled water at 2 mg/mL concentration, and used at 0.5 μg/mL.

3-(4,5-dimethylthiazol-2-yl)-2,5-diphenyltetrazolium bromide (MTT; Sigma-Aldrich, St. Louis, MO, USA) was made in bidistilled water at 25 mg/mL concentration and used at 5 mg/mL.

The powder of propidium iodide (PI, HiMedia Laboratories, Mumbai, India) was dissolved in bidistilled water in order to obtain the stock solution at 12.5 mg/mL concentration. Then, the PI work solution (50 μg/mL) was made with sodium citrate (37 mM), Triton X-100 (0.1% *v*/*v*; Lonza; Basel, Switzerland) in bidistilled water [29].

Annexin A1-mimetic peptide (Ac2-26; single letter sequence: AMVSEFLKQAWFIENEEQEYVQTVK; modifications: Ala-1 = N-terminal Ac) was purchased from Tocris Bioscience (Tocris Bioscience, part of Bio-Techne; Minneapolis, MN, USA), made in phosphate saline buffer (PBS) 1× at 1 mM concentration, and used at 1 μM as reported in [30].

Finally, t-Boc-Met-Leu-Phe (Boc1) and t-Boc-Phe-d-Leu-Phe-d-Leu-Phe (Boc2) were obtained from Tocris Bioscience (Tocris Bioscience, part of Bio-Techne; Minneapolis, MN, USA), made in PBS 1× at 2 mM concentration, and used at 100 μM, acting as pan-antagonists of FPR isoforms [31].

### 2.3. Cell Blocks

The cells, seeded into 100 mm dishes and treated for 24 h, were detached and centrifuged for 5 min at 335× *g*. After two washes with culture medium, the pellets were gently resuspended in neutral-buffered formalin with pH 7.2–7.4 (formaldehyde 4%, DIAPATH, Martinengo, BG, Italy) to fix them. Following the manufacturer’s instructions, cell blocks (CBs) were generated using the Shandon Cytoblock Cell Block Preparation System (Thermo Fisher Scientific; Waltham, MA, USA). The cytological material was spun in a falcon tube for 10 min at 524× *g*. After recovering the cellular pellets and discarding the liquid supernatant, Reagent 2 was added in a sufficient quantity and vortexed. Next, after adding a few drops of Reagent 1 to the insert well’s center, a Cytoblock cassette formed in Shandon Cytospin 4; each Cytofunnel was then closed with a Cytoclip. The mixed cell suspension was put into the Cytospin for 3 min at 524× *g* in the Cytofunnel. After centrifugation, the cassette was positioned horizontally with the funnel removed, and one or two drops of Reagent 1 were introduced to the cassette before its enclosure and normal processing in alcohol. A typical paraffin embedding station was used for the embedding procedure to obtain paraffin blocks. By passing the tips of tiny forceps through the holes beneath the board insert, the insert was extracted from the Cytoblock cassette during embedding. Ultimately, slices of 4 µm stained with hematoxylin and eosin (H&E) were made to confirm the presence of many live cells.

### 2.4. Immunohistochemistry

Serial 4 µm tissue sections were cut from the paraffin blocks using an ordinary microtome and mounted on TOMO^®^ IHC adhesive pretreated glass slides (Matsunami Glass Ind., Ltd., Osaka, Japan). One section was stained with H&E to evaluate the cells’ morphology, and the other slides were used for immunohistochemistry (IHC) investigation. IHC staining was performed with the fully automated Ventana Benchmark Ultra platform (Ventana Medical Systems Inc., Tucson, AZ, USA) with the OptiView DAB IHC Detection Kit per both manufacturers’ recommendations. The sections were incubated with primary antibodies against CD44 (rabbit monoclonal antibody, clone EP700Y, Sigma-Aldrich, St. Louis, MO, USA) and CD166 (mouse monoclonal, clone BP-C375, Vector Laboratories, Burlingame, CA, USA). Finally, the immunostained slides were digitalized with an Aperio AT2 digital pathology slide scanner at 10× (Leica Biosystems Nussloch GmbH, Heidelberger, Germany). The immunostaining level of these antibodies was scored semiquantitatively; the staining intensity was rated as negative, equivocal, or positive. Positive staining demonstrated the target marker’s distinct and readily appreciable presence, with a clear staining pattern in ≥10% of tumor cells. Positivity for CD166 and CD44 was visualized as brown membrane and/or cytoplasmic staining [Supplementary Figure S1, panels a, a’ and c, c’, respectively]. Negative staining indicated the absence of specific staining or staining considered artifactual, or ≤10% of tumor cells showing positive staining [Supplementary Figure S1, panels b, b’ and d, d’]. Equivocal staining indicated weak, ambiguous, or focal staining that did not meet the criteria for positive or negative staining [Supplementary Figure S1, panels e, e’]. Two pathologists independently scored the immunostaining level of these antibodies semiquantitatively [S.S. and G.I.]. In cases of discrepancy between the two pathologists, a third independent pathologist was consulted to reach a final consensus score.

### 2.5. Confocal Microscopy

The OSCC cell lines, were seeded on microscope cover glasses (3 × 10^4^ cells/well) and treated for 24 h; then, they were fixed in p-formaldehyde (4% *v*/*v* in PBS; Lonza; Basel, Switzerland) for 10 min., permeabilized with Triton X-100 (0.5% *v*/*v* in PBS 1×; Lonza; Basel, Switzerland) for 5 min, and finally blocked with goat serum (20% *v*/*v* in PBS 1×; Lonza; Basel, Switzerland) for 20 min. Next, these cells were incubated with antibodies against CD44 (rabbit monoclonal antibody, clone EP700Y, Sigma-Aldrich, St. Louis, MO, USA), CD166 (mouse monoclonal, clone BP-C375, Vector Laboratories, Burlingame, CA, USA), ANXA1 (rabbit polyclonal, 1:100; Thermo Fisher Scientific; Waltham, MA, USA), E-cadherin (mouse monoclonal, 1:500; BD Transduction Laboratories™; Franklin Lakes, NJ, USA), and N-cadherin (rabbit polyclonal, 1:100; Elabsciences, Houston, TX, USA) O/N at 4 °C. Finally, the staining with secondary antibodies, anti-mouse and anti-rabbit antibodies, respectively, for each antibody, and DAPI (1:1000; Thermo Fisher Scientific; Waltham, MA, USA) for the nuclei was performed for 2 h at room temperature (RT) in the dark. Confocal analysis was performed with Zeiss Confocal microscope LSM 510 (software version 4.0 SP1; Carl Zeiss MicroImaging GmbH, Jena, Germany) as described in [32], and CD44 and CD166 staining were performed with a TCS SP8 confocal microscope (software version 3.5.7.23225; Leica Microsystems, Wetzlar, Germany).

### 2.6. Wound-Healing Assay

The cellular lines were seeded in a 12-well plate at 1 × 10^5^ cells per well. After 24 h, cells reached 100% confluency, and a wound was produced on the monolayer by scraping the cells with a sterile plastic p10 pipette tip. Following washing with PBS 1×, the treatment (Ac2-26 1 μM, 5-AZA 100 μM or the growth medium as control) was added. Moreover, at each experimental condition, Mitomycin C (0.5 μg/mL, Sigma-Aldrich; St. Louis, MO, USA) was further added to ensure the blockage of mitosis. In the case of transfection with ANXA1’s siRNA, plated cells were transfected for 24 h with siRNAs, and then the wound was produced and treated as reported above where required. The wounded cells were photographed at 0 and 24 h using an Axiovert 5 microscope with an Axiocam 208 color camera (Carl Zeiss; Oberkochen, Germany). A 10× phase contrast objective was used. The values shown in the histograms represent the analysis obtained from the average of the measured distances (reported as µm) at different positions, for which some cells were selected on both sides of the wound.

### 2.7. Invasion Assay

Cell invasiveness was studied using a trans-well cell culture of 12 mm diameter and 8.0 fim pore size (Corning Incorporated; New York, NJ, USA), as previously described [33]. In the upper chamber, the cells were seeded, while in the lower one of each well, the treatments were added as established in the experimental conditions and as performed in the wound-healing assay. Mitomycin C (0.5 μg/mL, Sigma-Aldrich; St. Louis, MO, USA) was included to ensure the arrest of mitosis. After 24 h, each trans-well was washed twice with PBS 1x and fixed in p-formaldehyde (4% *v*/*v* in PBS 1×; Lonza; Basel, Switzerland) for 10 min and then in 100% methanol (PanReac Applichem; Darmstadt, Germany) for 20 min. Staining was performed with 0.5% crystal violet (crystal violet powder, Merck Chemicals, and distilled water at 20% methanol) for 30 min. The trans-wells were photographed using an Axiovert 5 microscope with an Axiocam 208 color camera (Carl Zeiss; Oberkochen, Germany). A 10× phase contrast objective was used. The histograms were obtained through analysis via cell counting of the number of cells present in the trans-well.

### 2.8. MTT Assay

The MTT assay was performed on CAL 27 and CAL33 cells. Briefly, cells were seeded in 96-well plates (5 × 10^3^ cells/well) and left in the culture medium for 24 h. Then, cells were treated with 5-AZA at concentrations of 25 μM, 100 μM and 200 μM for 24, 48 and 72 h. At the end of the treatment, 25 μL (5 mg/mL) of MTT (Sigma-Aldrich, St. Louis, MO, USA) was added in each well. After 3 h of incubation, 100 μL of DMSO (Sigma-Aldrich, St. Louis, MO, USA) was added to each well. Cells were incubated at RT on a shaker for 10 min to solubilize formazan salts. The optical density (OD) of each well was measured with a spectrophotometer (Multiskan Spectrum; Thermo Electron Corporation, Waltham, MA, USA) equipped with a 550 nm filter.

### 2.9. Flow Cytometry for Cell Cycle and Cell Death

CAL 27 and CAL33 cells were plated in 24-well plates (5 × 10^4^ cells/well) and analyzed for cell cycle and death [29]. In both cases, after treatment, cells were stained with PI (HiMedia Laboratories, Mumbai, India) working solution and analyzed by flow cytometry (FACScan cytometer, Becton-Dickinson, Franklin Lakes, NJ, USA). The percentage of dead cells was directly analyzed by the Cell Quest program, version 6.0, by which 1 × 10^5^ events were evaluated by separating necrotic nuclei from apoptotic ones and both from viable cells in FSC/SSC and FL2/count plots. For the assessment of cell cycle phases, viable cell samples were further analyzed through the ModFit LT analysis software, version 3.0.

### 2.10. Western Blotting

For protein analysis, different kinds of samples were processed. For total protein extracts, the cells were harvested directly with 1x gel-loading buffer (LB 1×) (50 mM Tris–Cl pH 6.8; 2% *w*/*v* SDS; 0.1% bromophenol blue; 10% (*v*/*v*) glycerol and 100 mM β-mercaptoethanol) and sonicated for 30 s, 9.9 s pulse on, and 9.9 s pulse off at an amplitude of 28% (Vibra-Cell Sonics; Sonics & materials, INC; Newtown, CT, USA). Furthermore, annexin A1 membrane-bound assay was performed as follows: cells were seeded into a 6-well plate (5 × 10^4^ cells/well) and after 24 h of treatment, they were washed in PBS 1× and incubated for 1 h with cold 2 mM PBS-EDTA solution. This membrane-bound (MB) ANXA1 supernatants were collected, clarified (centrifuged at 335× *g* for 5 min), and concentrated with centrifugal filter units (Amicon Ultra-0.5 mL, Merck Millipore; Darmstadt, Germany) according to both manufacturers’ recommendations. Additionally, for annexin A1 supernatant assay, CAL27 cells were plated into 6-well plates (5 × 10^4^ cells/well) and treated for 24 h in culture medium without FBS. The supernatants were collected, clarified (centrifuged at 335× *g* for 5 min), and concentrated with centrifugal filter units (Amicon Ultra-0.5 mL, Merck Millipore, Darmstadt, Germany) as per the kit instructions. Protein samples, lysed in LB1x, were examined by Sodium dodecyl sulphate-polyAcrylamide gel electrophoresis (SDS-PAGE). The proteins were then transferred electrophoretically to nitrocellulose blotting membranes (Amersham™ Protran™, Cytiva; Little Chalfont, UK) and subsequently blocked with 5% non-fat dry milk in TBS-Tween 20 (0.1% *v*/*v*). The proteins were visualized using the chemioluminescence detection system (Amersham™, Cytiva; Little Chalfont, UK) after O/N incubation at 4 °C with primary antibodies against ANXA1 (rabbit polyclonal, 1:10,000; Thermo Fisher Scientific; Waltham, MA, USA), GAPDH (mouse monoclonal 1:1000; Sigma-Aldrich; St. Louis, MO, USA), β-tubulin (mouse monoclonal, 1:2000; Cohesion Biosciences; London, UK), and then at RT with an appropriate secondary rabbit or mouse antibody for 1 h (1:10,000; Jackson ImmunoResearch; Philadelphia, PA, USA). The blots were exposed to Las4000 (GE Healthcare Life Sciences, Little Chalfont, UK), and the relative band intensities, expressed by optical densitometry (OD), were determined using ImageJ software 2.14.0 version (National Institutes of Health and the Laboratory for Optical and Computational Instrumentation; LOCI, University of Wisconsin, Madison, WI, USA).

### 2.11. RNA Extraction and Quantitative Real Time-Polymerase Chain Reaction (qRT-PCR)

Total CAL27 RNA was extracted using TRIzol reagent (Invitrogen, no. 15596018, Auckland, New Zealand) following the manufacturer’s instructions, and 1 µg of each RNA was reverse transcribed into cDNA with Transcriptor First Strand cDNA Synthesis Kit (Roche, Indianapolis, IN, USA). cDNAs were processed using Light Cycler 480 Syber Green I Master mix and primers for ANXA1 (Bio-Fab research; Rome, Italy) (5′-GCAAGAAGGTAGAGATAAAGACACT-3′, 3′-ACAAGTTTGACACTTCAGTAGGTT-5′) and Hypoxanthine Phosphoribosyltransferase 1 (HPRT1) (Bio-Fab research; Rome, Italy) (5′-GACCAGTCAACAGGGGACAT-3′, 3′-CCTGACCAAGGAAAGCAAAG-5′) following the manufacturer’s protocols. The mRNA levels of CAL27 cells were evaluated by real-time PCR using the Light Cycler 480 II instrument (Roche, Indianapolis, IN, USA) and they were analyzed using the Delta–Delta CT method [34].

### 2.12. siRNAs Transfection Death

The siRNA sequences against ANXA1 were purchased from Santa Cruz Biotechnology, Inc. (Dallas, TX, USA). Different concentrations of siRNA were tested for different times, and the final concentration was finally chosen and used at 100 nM. siRNA Oligo-Scrambled (Santa Cruz Biotechnology, Inc., Dallas, TX, USA) was used as control at the same concentration. These sequences were transfected using Transit (Mirius, TEMA RICERCA srl, Bologna, Italy), according to the manufacturer′s instructions. Cells were harvested 24 and 48 h after transfection and then processed according to the experimental plan.

### 2.13. Statistical Analysis

Data analyses and the statistical evaluation were carried out using Microsoft Excel and GraphPad Prism 6. The independent experiments were repeated at least three times in triplicate, and all results were shown as the mean ± standard deviation (SD). Regarding statistical analysis, the different groups were compared using one-way ANOVA with Tukey’s post hoc and two-tailed *t*-test, as appropriate. Differences were considered significant if *p* ≤ 0.05. The number of independent experiments and *p*-values are indicated in the figure legends.

## 3. Results

### 3.1. Different Phenotypes of CAL27 and CAL33 Cells in Presence of 5-AZA Treatment

**Figure 1:**
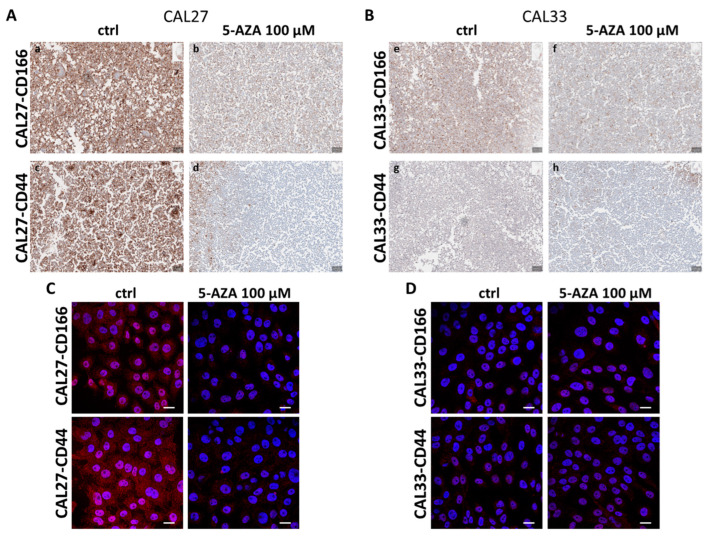
Characterization of CAL27 and CAL33 cells. (**A**) Representative images from IHC on sections from cell blocks of CAL27 cells treated or untreated for 24 h with 5-AZA 100 μM and immunostained with CD166 (panels a and b) and CD44 (panels c and d) antibodies. (**B**) Representative images from IHC on sections from cell blocks of CAL33 cells treated or untreated for 24 h with 5-AZA 100 μM and immunostained with CD166 (panels e and f) and CD44 (panels g and h) antibodies. The immunostained slides were digitalized with an Aperio AT2 digital pathology slide scanner. Magnification = 10×. Scale bar = 50 μm. The immunostaining level of these antibodies was scored semiquantitatively. The intensity of staining was rated as negative, equivocal, or positive, following the evaluation criteria reported in the Materials and Methods Section. Positivity for CD166 and CD44 was visualized as brown membrane and/or cytoplasmic staining. Representative images of immunofluorescence evaluation of CD166 and CD44 (both in red) on CAL27 (**C**) and CAL33 (**D**) cells after treatments with 5-AZA 100 μM for 24 h. Nuclei were stained with DAPI (blue) 1:1000 for 2 h at RT in the dark. Magnification = 63×. Scale bar = 10 μm. Data are representative of three independent experiments with similar results.

Firstly, we characterized two immortalized cell lines of the human tongue squamous cell carcinoma, CAL27 and CAL33, by IHC. We assessed CD166 and CD44 expression and their cellular localization on the sections obtained using the cell block technique. The immunostaining shown in Figure 1A,B revealed the basal differences between the two cell lines. In particular, CAL27 cells are characterized by strong positivity and well-defined membrane signal for the stemness markers CD166 and CD44 (Figure 1A, panels a and c). In the presence of 5-AZA at the concentration of 100 µM, as used in a previous study [15], the cells lost the expression of these stemness markers (Figure 1A, panels b and d). In contrast, in the CAL33 cells, the basal signal of both proteins appeared to be negative (Figure 1B, panels e and g), and, even after treatment, they continued to exhibit negative staining (Figure 1B, panels f and h). The treatment was performed for 24 h in order to study the variations in the protein expression and to avoid the potential cytotoxic effect of this agent that generally occurs over longer exposure time [35]. Furthermore, in order to confirm the IHC results, we perfomed IF staining with the same antibodies (Figure 1C for CAL27 and 1D for CAL33 cells).

### 3.2. In CAL27, 5-AZA Reduces Cell Motility and Induces MET

**Figure 2:**
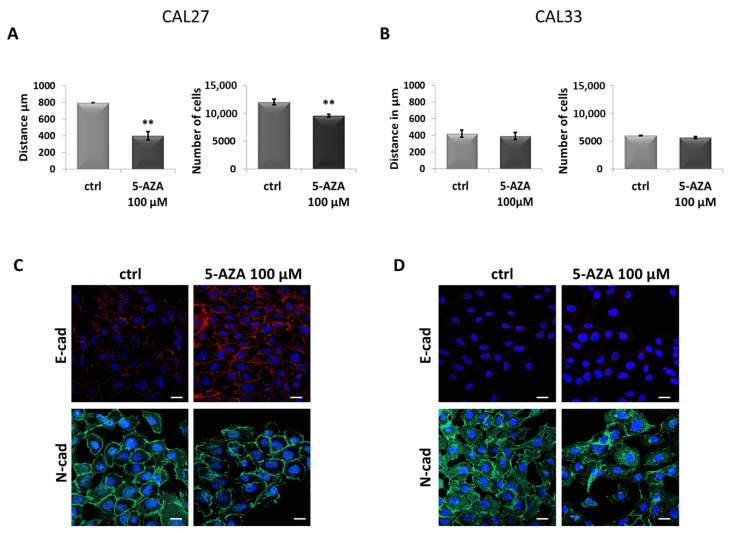
Analysis of cell motility and phenotype of CAL27 and CAL33 cells in the presence of 5-AZA. (**A**) Results from the migration and invasion assays, respectively, on CAL27 cells treated or untreated for 24 h with 5-AZA 100 μM. The histograms represent the analysis obtained. (**B**) Results from the migration and invasion assays, respectively, on CAL33 cells treated or untreated for 24 h with 5-AZA 100 μM. The histograms represent the analysis obtained. (**C**) Representative images of immunofluorescence evaluation of E-cadherin (red) and N-cadherin (green) on CAL27 cells after treatments with 5-AZA 100 μM for 24 h. Nuclei were stained with DAPI (blue) 1:1000 for 2 h at RT in the dark. Magnification = 63×. Scale bar = 10 μm. (**D**) Representative images of immunofluorescence evaluation of E-cadherin (red) and N-cadherin (green) on CAL33 cells after treatments with 5-AZA 100 μM for 24 h. Nuclei were stained with DAPI (blue) 1:1000 for 2 h at RT in the dark. Magnification = 63×. Scale bar = 10 μm. Data represent the mean of three independent experiments ± S.D. with similar results; error bars represent the S.D. ** *p* < 0.01 versus untreated control.

Once these cell lines were characterized, we studied how 5-AZA could influence CAL27 and CAL33 cell motility. Cell migration was analyzed by a wound-healing assay, and the capability of cell invasion was analyzed by coating matrigel on trans-wells, as reported in the Materials and Methods Section. As shown in Figure 2A, CAL27 migrated and invaded significantly more slowly in the presence of 5-AZA when compared to the untreated control. Moreover, with the same treatment, CAL33 did not show a change in cell motility in terms of migration and invasion (Figure 2B). In addition, to support these results, we characterized the cells according to their acquired phenotype after treatment. It is known that poorly aggressive and invasive features of tumor cells can be characterized by certain markers following a mesenchymal–epithelial transition (MET) [36]. Our confocal analysis confirmed these kinds of phenotypic changes on the CAL27 cell line treated with 5-AZA through the up-regulation of the E-cadherin protein as an epithelial marker and the downregulation of the N-cadherin prtotein as a mesenchymal one (Figure 2C). Interestingly, no significant results have been obtained for CAL33 cells (Figure 2D).

### 3.3. 5-AZA Induces Increased Expression of ANXA1 in CAL27 Cells

**Figure 3:**
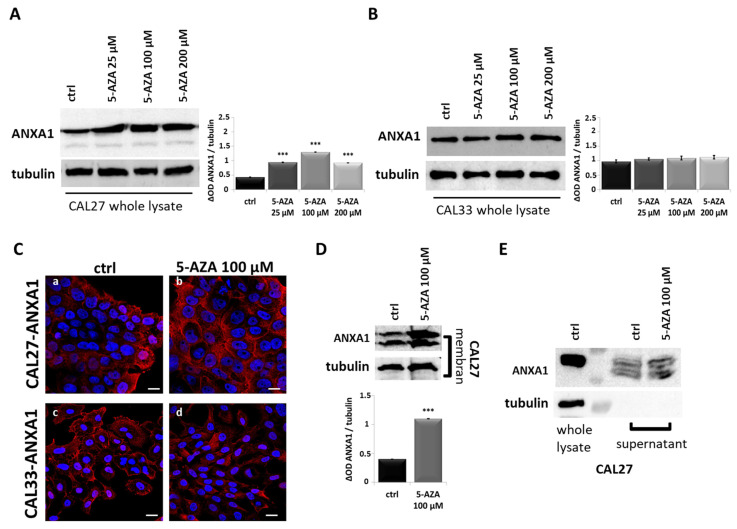
Analysis of ANXA1 expression in CAL27 and CAL33 cells. (**A**) ANXA1 protein levels were detected in CAL27 cells after treatment with increasing concentrations of 5-AZA (25 μM, 100 μM and 200 μM) for 24 h by Western blotting. Protein normalization was performed on tubulin levels, and the relative intensities of signals were expressed by OD, determined using ImageJ, and reported alongside. (**B**) ANXA1 protein levels were detected in CAL33 cells after treatment with increasing concentrations of 5-AZA for 24 h by Western blotting. Protein normalization was performed on tubulin levels, and the relative intensities of signals were expressed by OD, determined using ImageJ, and reported alongside. (**C**) Representative images of immunofluorescence evaluation of ANXA1 (red) on CAL27 (panels a,b) and CAL33 (panels c,d) after treatments with 5-AZA 100 μM. Nuclei were stained with DAPI (blue) 1:1000 for 2 h at RT in the dark. Magnification = 63×. Scale bar = 10 μm. (**D**) ANXA1 protein levels in CAL27 membrane and supernatant lysates (**E**) obtained from cells treated or untreated for 24 h with 5-AZA 100 μM. Protein normalization was performed on tubulin levels, and the relative intensities of signals were expressed by OD, determined using ImageJ, and reported alongside. In detail, in (**E**), the first experimental condition was the CAL27 whole lysate being used as a technical control in order to show the presence of tubulin protein exclusively in this sample and not in the supernatant counterparts. Data represent the mean of three independent experiments ± S.D. with similar results; error bars represent the S.D. *** *p* < 0.001 versus untreated control.

We treated both cell lines with increasing concentrations (25 µM, 100 µM and 200 µM) of 5-AZA for 24 h (Figure 3A,B). Figure 3A shows higher expression of ANXA1 in the CAL27 whole lysate at concentrations of 100 µM and 200 µM, while CAL33 did not show the same response (Figure 3B). In this regard, we also chose to continue the study with 5-AZA treatment at 100 µM because the cells maintained high viability, as reported in Supplementary Figure S2. The confocal analysis confirmed these data, highlighting the increased and well-structured ANXA1 fluorescence signal only in CAL27 after treatment (Figure 3C, panels a and b) compared to the CAL33 cells (Figure 3C, panels c and d). Furthermore, to define the effect of the drug on ANXA1 expression, we showed that the treatment also induced the increase in protein levels in the CAL27 membrane (Figure 3D) and supernatant lysates (Figure 3E), obtained as reported in the Materials and Methods Section. Finally, through a qRT-PCR analysis on CAL27 samples treated with 5-AZA at a concentration of 100 µM for 4 and 24 h, we excluded a direct positive regulation of ANXA1 transcript induction by this drug; rather, a slight decrese in its expression was observed (Supplementary Figure S3).

### 3.4. siRNA-Mediated ANXA1 Downmodulation Restores the CAL27 Aggressive Phenotype

**Figure 4:**
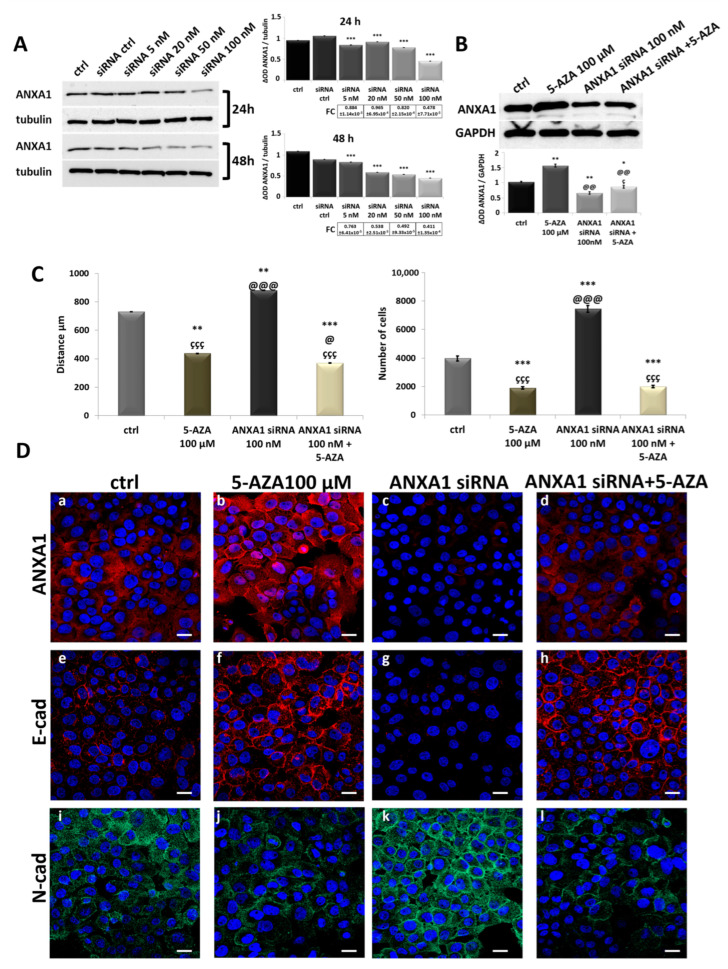
Analysis on CAL27 cells of the ANXA1 siRNA action alone and in combination with 5-AZA. (**A**) ANXA1 protein levels were detected in CAL27 cells after ANXA1 siRNA treatment at different concentrations at 24 and 48 h by Western blotting. Protein normalization was performed on tubulin levels, and the relative intensities of signals were expressed by OD, determined using ImageJ, and reported alongside. (**B**) ANXA1 protein levels were detected in CAL27 cells after ANXA1 siRNA treatment at 100 nM for 48 h and with 5-AZA 100 μM for 24 h, alone or in combination. Protein normalization was performed on GAPDH levels, and the relative intensities of signals were expressed by OD, determined using ImageJ, and reported alongside. (**C**) Results from the migration and invasion assays, respectively, on CAL27 cells treated or untreated with ANXA1 siRNA treatment at 100 nM for 48 h and with 5-AZA 100 μM for 24 h, alone or in combination. The histograms represent the analysis obtained. (**D**) Representative images of immunofluorescence evaluation of ANXA1 (red) (panels a–d), E-cadherin (red) (panels e–h), and N-cadherin (green) (panels i–l) on CAL27 treated or untreated with ANXA1 siRNA treatment at 100 nM for 48 h and with 5-AZA 100 μM for 24 h, alone or in combination. Nuclei were stained with DAPI (blue) 1:1000 for 2 h at RT in the dark. Magnification = 63×. Scale bar = 10 μm. Data represent the mean of three independent experiments ± S.D. with similar results; error bars represent the S.D. * *p* < 0.05; ** *p* < 0.01; *** *p* < 0.001 versus untreated control; @ *p* < 0.05; @@ *p* < 0.01; @@@ *p* < 0.001 versus 5-AZA; ç *p* < 0.05; ççç *p* < 0.001 versus ANXA1 siRNAs.

In order to prove the importance of the 5-AZA-mediated high expression of ANXA1 in CAL27 cell line, we studied the action of protein downmodulation assessed by siRNAs. We tested the protein reduction using ANXA1 siRNAs at different concentrations at 24 and 48 h (Figure 4A). Thus, we chose the siRNA concentration of 100 nM at 24 h as the optimal condition. Firstly, we evaluated whether 5-AZA was able to partially restore the high levels of ANXA1, although protein reduction was provoked by siRNAs (Figure 4B). Then, we studied the resulting behavior in terms of motility and MET marker expression. Indeed, following protein downmodulation, we observed the restoration of the aggressive phenotype as shown by the significant rise in cell migration/invasion compared to the untreated control (Figure 4C). However, in the experimental condition with the combination siRNAs and 5-AZA, the latter continued to perform its action, slowing down cell motility (Figure 4C). Moreover, thanks to confocal microscopy, the aggressive phenotype acquired by ANXA1 silencing was highlighted with a decrease in E-cadherin and increase in N-cadherin expressions (Figure 4D, panels g and k, respectively). Finally, when 5-AZA was added to the previously siRNA-transfected cells, the expression of the ANXA1 protein was slightly increased (Figure 4D, panel d) compared to the condition with siRNAs alone (Figure 4D, panels b and c). In detail, the cells, when silenced and then treated with 5-AZA, showed a behavior more similar to the counterpart treated exclusively with the drug, with an increase in E-cadherin and an opposite N-cadherin expression pattern (Figure 4D, panels h and l, respectively).

### 3.5. Exogenous Ac2-26 Reduces CAL27 Motility by Acquiring a Less Aggressive Phenotype

**Figure 5:**
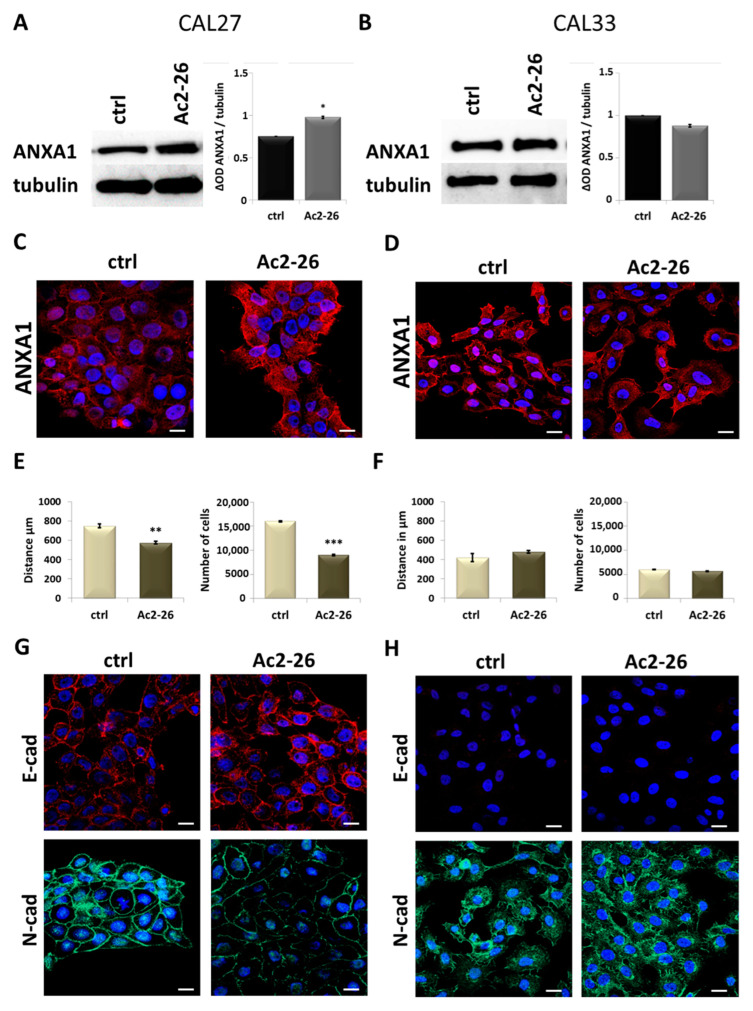
Study of the effect of Ac2-26 on CAL27 and CAL33 cells. (**A**) ANXA1 protein levels were detected in CAL27 cells after 24 h of Ac2-26 1 μM treatment by Western blotting. Protein normalization was performed on tubulin levels, and the relative intensities of signals were expressed by OD, determined using ImageJ, and reported alongside. (**B**) ANXA1 protein levels were detected in CAL33 cells after 24 h of Ac2-26 1 μM treatment by Western blotting. Protein normalization was performed on tubulin levels, and the relative intensities of signals were expressed by OD, determined using ImageJ, and reported alongside. (**C**) Representative images of immunofluorescence evaluation of ANXA1 (red) on CAL27 cells after treatment with Ac2-26 1 μM for 24 h. Nuclei were stained with DAPI (blue) 1:1000 for 2 h at RT in the dark. Magnification = 63×. Scale bar = 10 μm. (**D**) Representative images of immunofluorescence evaluation of ANXA1 (red) on CAL33 cells after treatment with Ac2-26 1 μM for 24 h. Nuclei were stained with DAPI (blue) 1:1000 for 2 h at RT in the dark. Magnification = 63×. Scale bar = 10 μm. (**E**) Results from the migration and invasion assays, respectively, on CAL27 cells treated or untreated for 24 h with Ac2-26 1 μM. The histograms represent the analysis obtained. (**F**) Results from the migration and invasion assays, respectively, on CAL33 cells treated or untreated for 24 h with Ac2-26 1 μM. The histograms represent the analysis obtained. (**G**) Representative images of immunofluorescence evaluation of E-cadherin (red) and N-cadherin (green) on CAL27 cells after treatment with Ac2-26 1 μM for 24 h. Nuclei were stained with DAPI (blue) 1:1000 for 2 h at RT in the dark. Magnification = 63×. Scale bar = 10 μm. (**H**) Representative images of immunofluorescence evaluation of E-cadherin (red) and N-cadherin (green) on CAL33 cells after treatment with Ac2-26 1 μM for 24 h. Nuclei were stained with DAPI (blue) 1:1000 for 2 h at RT in the dark. Magnification = 63×. Scale bar = 10 μm. Data represent the mean of three independent experiments ± S.D. with similar results; error bars represent the S.D. * *p* < 0.05; ** *p* < 0.01; *** *p* < 0.001 versus untreated control.

In the tumor microenvironment, the exogenous form of ANXA1 has been shown to stimulate the cellular components [34]. Particularly, the N-terminal mimetic peptide of ANXA1, Ac2-26, is able to induce similar physiopathological effects of this protein [37,38]. In this study, we assessed these effects on CAL27 and CAL33 cells by Ac2-26 administration, used at 1 μM, based on previous studies [29,30]. In this case, we observed an increase in ANXA1 expression by Western blot (Figure 5A) and confocal microscopy analysis (Figure 5C) in CAL27 cells. No significant differences were found in CAL33 counterparts (Figure 5B,D). The investigation of cell motility revealed a significant reduction in migration and invasion rates in CAL27 cell lines (Figure 5E), while no differences were detected in CAL33 (Figure 5F). Moreover, to support the similar effects of exogenous ANXA1 and 5-AZA, our analyses further focused on MET marker expression. Also, in this case, only in CAL27, in the presence of Ac2-26, the expression of E-cadherin increased with a subsequent decrease in N-cadherin (Figure 5G).

### 3.6. ANXA1 Effects Are Triggered by FPR

**Figure 6:**
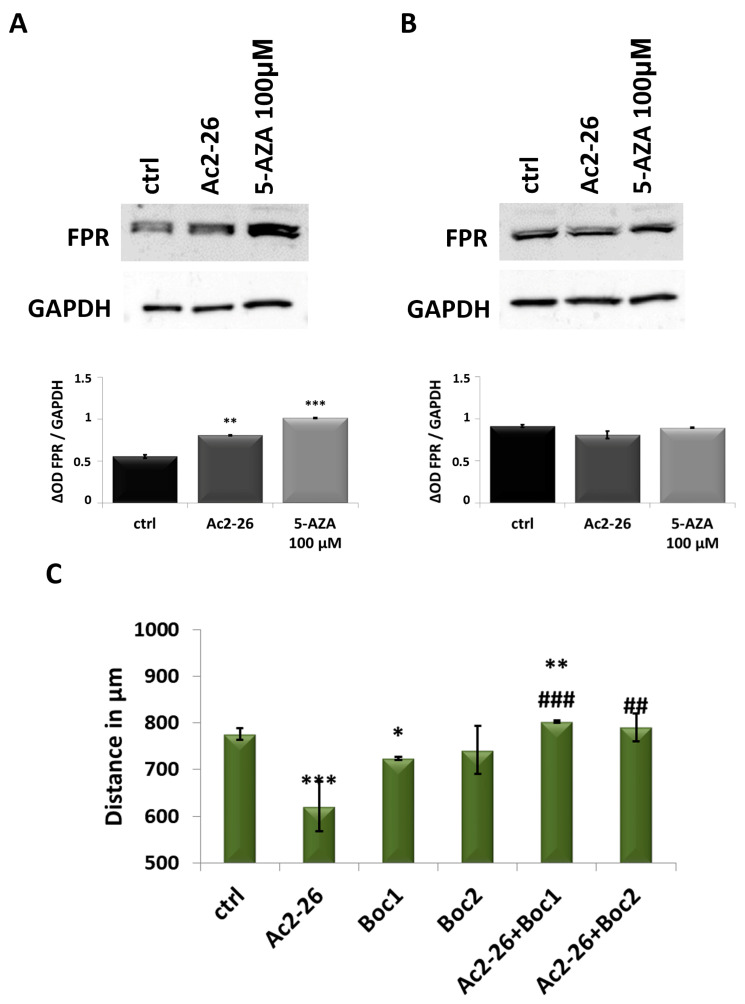
Investigation of ANXA1 action on FPR of CAL27 and CAL33 cells. (**A**) FPR protein levels were detected in CAL27 cells after 24 h of Ac2-26 1 μM and 5-AZA 100 μM treatments by Western blotting. Protein normalization was performed on GAPDH levels, and the relative intensities of signals were expressed by OD, determined using ImageJ, and reported alongside. (**B**) FPR protein levels were detected in CAL33 cells after 24 h of Ac2-26 1 μM and 5-AZA 100 μM treatments by Western blotting. Protein normalization was performed on GAPDH levels, and the relative intensities of signals were expressed by OD, determined using ImageJ, and reported along-side. (**C**) Results from the migration assay on CAL27 cells treated for 24 h with Ac2-26 1 μM, Boc1, and Boc2 100 μM. The histograms represent the analysis obtained. Data represent the mean of three independent experiments ± S.D. with similar results; error bars represent the S.D. * *p* < 0.05; ** *p* < 0.01; *** *p* < 0.001 versus untreated control; ## *p* < 0.01; ### *p* < 0.001 versus Ac2-26.

Once the exogenous ANXA1 effects were evaluated, we assessed FPR expression, referred to as its better-characterized receptor [16], on CAL27 and CAL33 cells by Western blot analysis (Figure 6A,B). In CAL27, the expression of FPR increased in the presence of Ac2-26 and 5-AZA treatments for 24 h (Figure 6A), whereas no difference was observed in the other cell line (Figure 6B). Thus, we examined the migration ability of CAL27 treated with t-Boc-Met-Leu-Phe (Boc1) and t-Boc-Phe-d-Leu-Phe-d-Leu-Phe (Boc2) at 100 μM, which act as pan-antagonists of FPR isoforms [31]. The results in Figure 6C show that both antagonists of FPR did not induce a significant change in cell speed when compared to untreated cells. Interestingly, when these compounds were associated with Ac2-26, CAL27 cells migrated more rapidly than the experimental condition with Ac2-26 alone.

### 3.7. ANXA1 Action Is Highlighted by Ac2-26 Use

**Figure 7:**
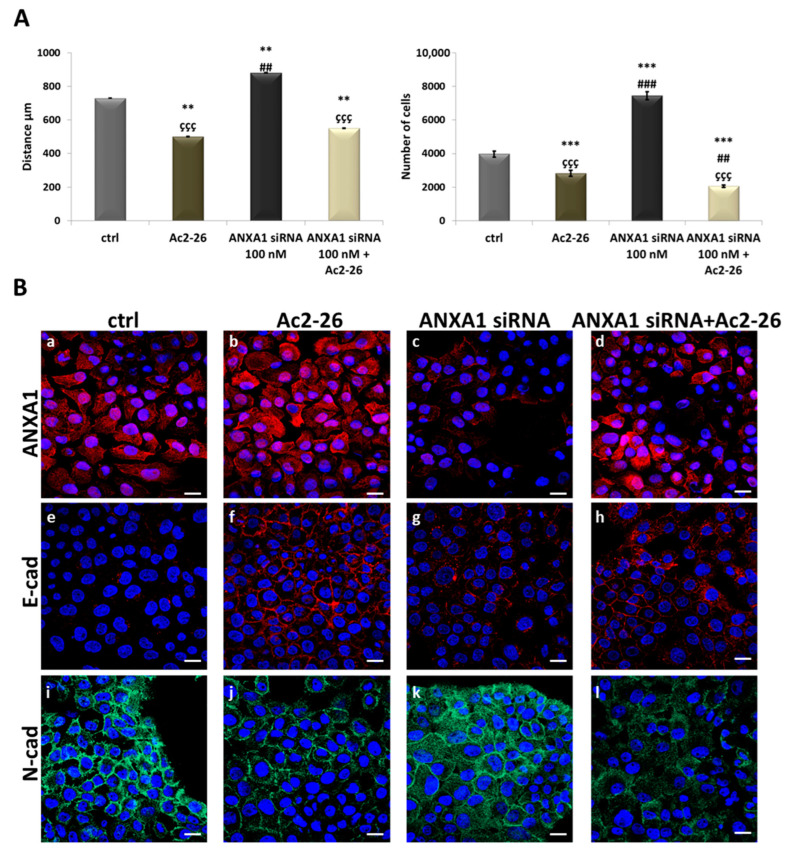
Investigation of Ac2-26 action on silenced CAL27 cells. (**A**) Results from the migration and invasion assays, respectively, on CAL27 cells treated or untreated with ANXA1 siRNA treatment at 100 nM for 48 h and with Ac2-26 1 μM for 24 h, alone or in combination. The histograms represent the analysis obtained. (**B**) Representative images of immunofluorescence evaluation of ANXA1 (red) (panels a–d), E-cadherin (red) (panels e–h), and N-cadherin (green) (panels i–l on CAL27 cells treated or untreated with ANXA1 siRNA treatment at 100 nM for 48 h and with Ac2-26 1 μM for 24 h, alone or in combination. Nuclei were stained with DAPI (blue) 1:1000 for 2 h at RT in the dark. Magnification = 63×. Scale bar = 10 μm. Data represent the mean of three independent experiments ± S.D. with similar results; error bars represent the S.D. ** *p* < 0.01; *** *p* < 0.001 versus untreated control; ## *p* < 0.01; ### *p* < 0.001 versus Ac2-26; ççç *p* < 0.001 versus ANXA1 siRNAs.

In order to continue our investigation on ANXA1’s role in OSCCs and, in particular, to deepen our understanding of its exogenous form, we evaluated the influence of Ac2-26 on ANXA1-downmodulated CAL27 by analyzing cell motility and the change in MET marker expression. From the analysis of migration and invasion assays, we observed that, even in this case, the subsequent exogenous administration of Ac2-26 to the previously silenced cells reduced both cell migration speed and the number of invading cells, when compared to the experimental condition with only siRNAs, as shown in Figure 7A. Finally, through immunofluorescence images, we analyzed the expression of MET markers. Following the downmodulation of ANXA1, which displayed, as previously mentioned (Figure 4D, panels g and k), the acquisition of a more aggressive phenotype (Figure 7B, panels g and k), the subsequent administration of Ac2-26 led to an increase in the expression of E-cadherin, with a reduction in N-cadherin expression (Figure 7B, panels h and l, respectively). This phenotype reversion appeared markedly similar to CAL27 cells treated with Ac2-26 alone (Figure 7B, panels f and j, respectively).

## 4. Discussion

Progresses in treating OSCCs are limited both due to lack of information on diagnostic and prognostic tumor markers, and due to pharmacological treatment resistance and relapses; the latter is mainly due to the intrinsic cellular complexity and heterogeneity of the tumor [39,40]. Recent studies have demonstrated the presence of distinct tumor cell subpopulations within the HNSCC that are involved in responses to anti-cancer treatment, which is often unsuccessful [39,41]. Therefore, for an anti-cancer strategy to be effective, it should be personalized, considering this heterogeneity, and should be based on targeted marker therapy.

In this research, we studied two cell lines, CAL27 and CAL33, derived from human tongue squamous cell carcinoma. In detail, CAL27 cells are known to have been isolated from a poorly differentiated squamous cell carcinoma and are referred to as a more aggressive cell line than CAL33 cells, which are derived from a moderately differentiated squamous cell carcinoma and are endowed with greater chemosensitivity [28]. In our work, these lines exhibited specific protein expression patterns that should be considered as OSCC subpopulations, suggesting the presence of heterogeneous features in this model with different basal characteristics as indicated by the diverse CD44 and CD166 expression levels. Indeed, these proteins have been identified in several works as markers of cancer stem cells (CSCs) in epithelial cancers and HNSCC, being involved in different cellular functions [42]. As Yan et al. reported [43], CD166 is a marker currently used for detection of the CSCs in the HNSCC plasma membrane, and its altered expression is associated with tumor progression [44,45]. Moreover, CSCs are also well characterized by CD44 expression correlating with radio- and chemoresistance [46]. In order to support these findings, we relied on the concept that tumor cells, undergoing the epithelial-to-mesenchymal transition (EMT), dedifferentiate in a partially and transiently manner. This change in behavior begins with a modification in gene expression and the acquisition of a stem cell phenotype for propagation and self-perpetuation [47].

Our findings revealed that CAL27 cells exhibit a stemlike phenotype with high expression levels of CD44 and CD166, under basal conditions, and these markers are lost by treatment with the 5-AZA. In contrast, the lack of the expression of the above-mentioned markers on CAL33 cells explains the lack of response to 5-AZA. In this context, this drug can be used as an adjuvant therapeutic strategy capable of inducing cell reprogramming, since it is known to affect epigenetic alterations that lead to a reversion of the cell aggressive phenotype [48]. The experimental timeframe we chose is in line with what it has been reported in the literature, since the cytotoxic effect of 5-AZA is associated with its metabolism and manifests itself long after its incorporation into RNA (RNA:DNA incorporation ratio 65:35) [14,49,50]. For this reason, several in vitro studies report a cut-off time of 24 h for treatment with 5-AZA in order to assess the dysregulation of certain proteins of interest [9,35].

Here, we reported that a 5-AZA regimen significantly distresses the cellular processes involved in OSCC tumor progression, like cellular motility, meaning migration and invasion. In addition, it contributes to the reversion of the “cadherin switch” [51], with the restoration of E-cadherin, an epithelial marker, and the consequent downregulation of the counterpart, N-cadherin, promoting the acquisition of a less aggressive phenotype in CAL27 cells. The absence of effects on CAL33 cells highlights how different basal features can be crucial for sensitivity to the drug.

Our research has focused on ANXA1, a protein that has a dual role in cancer, acting both as an oncosuppressor and as an oncogene, depending on the cellular context and the type of tumor. Indeed, it has been reported that as an oncogene, its overexpression correlates with tumor progression by promoting malignant pathways and drug resistance, as described in several models, such as pancreatic cancer [52], melanoma [53] and gastric cancer [54]. Furthermore, in the case of antitumoral action, ANXA1 is involved in the inhibition of cell proliferation, promotion of apoptosis, and reduction of cell motility, as shown in breast cancer (triple negative) [55] and prostate cancer [56].

In OSCCs, ANXA1 has been suggested as a prognostic biomarker based on several clinical studies [20,57] reporting that its expression is markedly downregulated and correlated with the pathological differentiation grade in OSCC patients [19,58].

To date, few studies have addressed the underlying mechanisms responsible for the loss of ANXA1 expression in this type of cancer. Possible reasons could be mutations involved in the regulation and coding of the ANXA1 gene, genomic deletions, epigenetic mutations, miRNA-mediated repression, interaction and/or alteration of proteins that can regulate its transcription, post-translational modifications involved in protein degradation, increased degradation via the proteasome, and defects in protein transport or localization [59]. In this case, the lack of changes in ANXA1 mRNA levels suggests the involvement of intermediate pathways as the possible mechanism through which 5-AZA affects protein expression. Indeed, this drug was also able to partially restore the ANXA1 levels, despite silencing, and its impact was stronger than the siRNA downstream effect.

Interestingly, in our work, the absence of response of CAL33 to the alteration of ANXA1 levels in response to 5-AZA, unlike CAL27, whose protein markedly increases due to the drug, can be further explained by our previous characterization, which could be correlated with the origin of these cells. Furthermore, alongside its effects on ANXA1 expression, 5-AZA appeared to notably reduce the rate of migration and invasion through MET, which is the reverse process of EMT.

Through loss-of-function experiments, we established that ANXA1 acts as a tumor suppressor in OSCCs, particularly in CAL27 cells with a stem cell phenotype, because its downmodulation restored the motility and resulted in a more aggressive phenotype. Thus, our data also confirmed what Wan et al. already showed, namely that forced expression of ANXA1 inhibited cell proliferation and invasion, contributing to MET [21]. Interestingly, the biological rescue we assessed here does not require the use of molecular biology techniques to regulate protein expression, but rather, 5-AZA is a maker of these events. This latter finding represents the main goal of this work and can pave the way for broader relevant clinical implications. In detail, our results showed that the 5-AZA-mediated ANXA1 overexpression affects only poorly differentiated and more aggressive cells, indicating its role in tumor prognosis and in the definition of a targeted therapeutic plan. However, further studies are required regarding the molecular pathways through which ANXA1 exerts its role in OSCCs and the mechanism through which 5-AZA regulates this protein.

5-AZA is a well-known demethylating agent that acts on the regulation of a plethora of proteins. Thus, the investigation of cell pathways that could be affected by this drug represents, in our opinion, a very appealing perspective aimed at extensively exploring the action of 5-AZA, whether or not it is dependent on the protein of our interest. However, in this study, the analysis of the correlation between this agent and ANXA1, which is demonstrated for the first time, establishes a solid foundation in the context of the need for a targeted therapeutic adjuvant approach to improve OSCC management.

Interestingly, in this work, we showed that ANXA1 in CAL27 cells is also externalized in the presence of 5-AZA. The potential role of ANXA1 in the tumor microenvironment, investigated through the administration of Ac2-26, which is known as an ANXA1 mimetic peptide, highlighted the slowdown of cell motility and the consequent reversion of the cell aggressive phenotype exclusively in CAL27 cells and confirmed the crucial role of this protein, even as an external actor, but only in cases of poorly differentiated stemlike phenotype. It is well known that the secreted form of ANXA1, as an autocrine–paracrine factor, interacts with the FPR receptor family members, as largely described in both physiological and pathological models [34]. However, this protein was also reported to stimulate both FPR1 and 2, triggering anti-inflammatory pathways [23]. Interestingly, it has been reported that the neutrophil-derived antimicrobial peptide LL-37, which mediates cell chemotaxis as a ligand of FPRs in an anti-inflammatory context, might act as a tumor suppressor in OSCCs. Indeed, LL-37 is downmodulated and correlated with poor differentiation and increased metastasis in this kind of tumor [60]. So, we observed different roles of LL-37 and ANXA1 that are not entirely in accordance with the conventional function of FPRs as their receptors, which is generally associated with the promotion of tumor progression in terms of cell motility and resistance [34]. However, previous studies have shown the importance of ANXA1-FPR1 signaling axis in chemotherapy-induced antitumor immune responses [61].

Here, we also showed that 5-AZA can increase the expression of FPR in CAL27 cells, which probably indicates that increased ANXA1 expression activates and interacts, in an autocrine manner, with its receptor, thereby creating a positive loop aimed at enhancing its own activity. This kind of loop was further enhanced, as shown by Ac2-26, which also induced an increase in ANXA1. This feature was already reported in [62], showing that Ac2-26-treated Hep-2 cells exhibited increased ANXA1 expression. Moreover, the latter study highlighted the role of the ANXA1/FPR2 axis in laryngeal cancer, specifically defining ANXA1 as a key regulator of tumor growth and metastasis through paracrine mechanisms occurring in the tumor microenvironment.

Interestingly, the lack of this protein in more aggressive and resistant OSCC forms could require the induction of the levels/effects of this protein to make tumors more responsive to therapy. It is reasonable to hypothesize a model in which paracrine ANXA1, derived from non-tumoral cells, could exert these kinds of oncosuppressor effects on cells where its expression is weak, particularly in tumors with a major stem cell component. In this scenario, the immune system is known to play a crucial role in the regulation of tumor survival and is one of the most described system in which ANXA1 carries out its typical actions. Indeed, neutrophils, known as the immune counterpart in which ANXA1/FPR2 colocalization was previously detected in laryngeal cancer, may be considered the main cells that secrete the protein into the tumor microenvironment, exerting anti-inflammatory/antitumoral effects in a paracrine manner through interaction with the FPR receptor [62]. Moreover, it is now well recognized that inflammation, including that caused by alcohol/smoking abuse, is a risk factor for OSCCs. Indeed, it is evident that inflammatory cells have powerful effects on tumor development by acting both as promoters of malignancy [63] and of tumor progression by inducing the growth, migration, and differentiation of all cell types in the tumor microenvironment [64]. Thus, the modulation of exogenous ANXA1 may also serve as an oncosuppressor actor due to its anti-inflammatory effects in preventing OSCC progression. In this regard, in addition to the investigation of ANXA1/FPRs axis in terms of cell motility, it would be interesting to assess the means by which the triggered pathway affects the activity of inflammatory actors in future works. In particular, it would be reasonable to study the response of key cell populations of the immune system in the broader context of the organization of the complex tumor microenvironment.

## 5. Conclusions

In the present in vitro work, the effects of 5-AZA on immortalized OSCC tongue cells were investigated. This epigenetic therapy regimen significantly affects the OSCC tumor progression by reverting the aggressive phenotype only on CAL27 cells with stemlike features. Furthermore, we showed that the expression of ANXA1 was increased in 5-AZA-reprogrammed CAL27 cells acquiring a less aggressive phenotype. To the best of our knowledge, for the first time, this drug has been studied in correlation with ANXA1 protein since the possible targets involved in OSCCs were never explored in 5-AZA treatment. Additionally, the increased secreted form of ANXA1 is able to activate FPR by inducing the slowdown of tumor progression. This 5-AZA-mediated loop allowed us to suggest that the induction of ANXA1 expression/action from the other cell components of the microenvironment can revert the tumor cell phenotype. Taken together, this information provides evidence to consider ANXA1 as a potential marker for OSCCs as well as a novel therapeutic target. However, it can be useful to specify that there is a non-exclusive action of 5-AZA via ANXA1 and, vice versa, a non-exclusive activity of ANXA1 on the cell effects we found. Indeed other molecular pathways are surely involved in the complex process of tumor progression. Moreover, the possible mechanism of action of 5-AZA, whether dependent on ANXA1 or not, and, on the other hand, all the potential activities of this protein (i.e., tumor progression, tumor microenvironment and interaction between tumor and other cell populations) need to be further addressed. Nevertheless, our findings fit as an interesting new piece in the large and complex mosaic of knowledge about OSCCs and the ever-increasing necessity to focus on targeted therapy.

## Data Availability

The data presented in this study are available upon request from the corresponding author.

## Supplementary Materials

The following supporting information can be downloaded at https://www.mdpi.com/article/10.3390/cancers17071058/s1, Figure S1: Representative images for IHC quantification of CD44 and CD166 on CAL27 (A) and CAL33 (B) cells. Figure S2: Analysis of 5-AZA effects on cell death and cycle of CAL27 and CAL33 cells. Figure S3: Analysis of ANXA1 mRNA expression in CAL27.

## References

[1] Siegel R.L., Giaquinto A.N., Jemal A. (2024). Cancer statistics, 2024. CA Cancer J. Clin..

[2] Barsouk A., Aluru J.S., Rawla P., Saginala K., Barsouk A. (2023). Epidemiology, Risk Factors, and Prevention of Head and Neck Squamous Cell Carcinoma. Med. Sci..

[3] Pulte D., Brenner H. (2010). Changes in survival in head and neck cancers in the late 20th and early 21st century: A period analysis. Oncologist.

[4] Kałafut J., Czerwonka A., Anameriç A., Przybyszewska-Podstawka A., Misiorek J.O., Rivero-Müller A., Nees M. (2021). Shooting at Moving and Hidden Targets-Tumour Cell Plasticity and the Notch Signalling Pathway in Head and Neck Squamous Cell Carcinomas. Cancers.

[5] Feller G., Khammissa R.A.G., Ballyram R., Beetge M.M., Lemmer J., Feller L. (2023). Tumour Genetic Heterogeneity in Relation to Oral Squamous Cell Carcinoma and Anti-Cancer Treatment. Int. J. Environ. Res. Public Health.

[6] Huang S.H., O’Sullivan B. (2017). Overview of the 8th Edition TNM Classification for Head and Neck Cancer. Curr. Treat. Options Oncol..

[7] Zerp S.F., Stoter T.R., Hoebers F.J., van den Brekel M.W., Dubbelman R., Kuipers G.K., Lafleur M.V., Slotman B.J., Verheij M. (2015). Targeting anti-apoptotic Bcl-2 by AT-101 to increase radiation efficacy: Data from in vitro and clinical pharmacokinetic studies in head and neck cancer. Radiat. Oncol..

[8] Adamska A., Elaskalani O., Emmanouilidi A., Kim M., Abdol Razak N.B., Metharom P., Falasca M. (2018). Molecular and cellular mechanisms of chemoresistance in pancreatic cancer. Adv. Biol. Regul..

[9] Taylor S.M. (1993). 5-Aza-2′-deoxycytidine: Cell differentiation and DNA methylation. Leukemia.

[10] Abbey D., Seshagiri P.B. (2013). Aza-induced cardiomyocyte differentiation of P19 EC-cells by epigenetic co-regulation and ERK signaling. Gene.

[11] Hervouet E., Cheray M., Vallette F.M., Cartron P.F. (2013). DNA methylation and apoptosis resistance in cancer cells. Cells.

[12] Soengas M.S., Capodieci P., Polsky D., Mora J., Esteller M., Opitz-Araya X., McCombie R., Herman J.G., Gerald W.L., Lazebnik Y.A. (2001). Inactivation of the apoptosis effector Apaf-1 in malignant melanoma. Nature.

[13] Zhang C., Li H., Zhou G., Zhang Q., Zhang T., Li J., Zhang J., Hou J., Liew C.T., Yin D. (2007). Transcriptional silencing of the TMS1/ASC tumour suppressor gene by an epigenetic mechanism in hepatocellular carcinoma cells. J. Pathol..

[14] Cihák A., Vesely J., Skoda J. (1985). Azapyrimidine nucleosides: Metabolism and inhibitory mechanisms. Adv. Enzym. Regul..

[15] Hodjat M., Jourshari P.B., Amirinia F., Asadi N. (2022). 5-Azacitidine and Trichostatin A induce DNA damage and apoptotic responses in tongue squamous cell carcinoma: An in vitro study. Arch. Oral Biol..

[16] Biktasova A., Hajek M., Sewell A., Gary C., Bellinger G., Deshpande H.A., Bhatia A., Burtness B., Judson B., Mehra S. (2017). Demethylation Therapy as a Targeted Treatment for Human Papillomavirus-Associated Head and Neck Cancer. Clin. Cancer Res..

[17] Pan C., Issaeva N., Yarbrough W.G. (2018). HPV-driven oropharyngeal cancer: Current knowledge of molecular biology and mechanisms of carcinogenesis. Cancers Head Neck.

[18] Tan Y., Wang Z., Xu M., Li B., Huang Z., Qin S., Nice E.C., Tang J., Huang C. (2023). Oral squamous cell carcinomas: State of the field and emerging directions. Int. J. Oral Sci..

[19] Zhu D.W., Yang X., Yang C.Z., Ma J., Liu Y., Yan M., Wang L.Z., Li J., Zhang C.P., Zhang Z.Y. (2013). Annexin A1 down-regulation in oral squamous cell carcinoma correlates to pathological differentiation grade. Oral Oncol..

[20] Lin C.Y., Jeng Y.M., Chou H.Y., Hsu H.C., Yuan R.H., Chiang C.P., Kuo M.Y. (2008). Nuclear localization of annexin A1 is a prognostic factor in oral squamous cell carcinoma. J. Surg. Oncol..

[21] Wan Y.M., Tian J., Qi L., Li L.M., Xu N. (2017). ANXA1 affects cell proliferation, invasion and epithelial-mesenchymal transition of oral squamous cell carcinoma. Exp. Ther. Med..

[22] Foo S.L., Yap G., Cui J., Lim L.H.K. (2019). Annexin-A1—A Blessing or a Curse in Cancer?. Trends Mol. Med..

[23] Zhuang Y., Wang L., Guo J., Sun D., Wang Y., Liu W., Xu H.E., Zhang C. (2022). Molecular recognition of formylpeptides and diverse agonists by the formylpeptide receptors FPR1 and FPR2. Nat. Commun..

[24] Sheikh M.H., Solito E. (2018). Annexin A1: Uncovering the Many Talents of an Old Protein. Int. J. Mol. Sci..

[25] Guo C., Liu S., Sun M.Z. (2013). Potential role of Anxa1 in cancer. Future Oncol..

[26] Hagihara T., Kondo J., Endo H., Ohue M., Sakai Y., Inoue M. (2019). Hydrodynamic stress stimulates growth of cell clusters via the ANXA1/PI3K/AKT axis in colorectal cancer. Sci. Rep..

[27] Raulf N., Lucarelli P., Thavaraj S., Brown S., Vicencio J.M., Sauter T., Tavassoli M. (2018). Annexin A1 regulates EGFR activity and alters EGFR-containing tumour-derived exosomes in head and neck cancers. Eur. J. Cancer.

[28] Gioanni J., Fischel J.L., Lambert J.C., Demard F., Mazeau C., Zanghellini E., Ettore F., Formento P., Chauvel P., Lalanne C.M. (1988). Two new human tumor cell lines derived from squamous cell carcinomas of the tongue: Establishment, characterization and response to cytotoxic treatment. Eur. J. Cancer Clin. Oncol..

[29] Novizio N., Belvedere R., Morretta E., Tomasini R., Monti M.C., Morello S., Petrella A. (2022). Role of Intracellular and Extracellular Annexin A1 in MIA PaCa-2 Spheroids Formation and Drug Sensitivity. Cancers.

[30] Dalli J., Montero-Melendez T., McArthur S., Perretti M. (2012). Annexin A1 N-terminal derived Peptide ac2-26 exerts chemokinetic effects on human neutrophils. Front. Pharmacol..

[31] Ye R.D., Boulay F., Wang J.M., Dahlgren C., Gerard C., Parmentier M., Serhan C.N., Murphy P.M. (2009). International Union of Basic and Clinical Pharmacology. LXXIII. Nomenclature for the formyl peptide receptor (FPR) family. Pharmacol. Rev..

[32] Proto M.C., Fiore D., Piscopo C., Franceschelli S., Bizzarro V., Laezza C., Lauro G., Feoli A., Tosco A., Bifulco G. (2017). Inhibition of Wnt/β-Catenin pathway and Histone acetyltransferase activity by Rimonabant: A therapeutic target for colon cancer. Sci. Rep..

[33] Belvedere R., Novizio N., Pessolano E., Tosco A., Eletto D., Porta A., Campiglia P., Perretti M., Filippelli A., Petrella A. (2020). Heparan sulfate binds the extracellular Annexin A1 and blocks its effects on pancreatic cancer cells. Biochem. Pharmacol..

[34] Novizio N., Belvedere R., Pessolano E., Tosco A., Porta A., Perretti M., Campiglia P., Filippelli A., Petrella A. (2020). Annexin A1 Released in Extracellular Vesicles by Pancreatic Cancer Cells Activates Components of the Tumor Microenvironment, through Interaction with the Formyl-Peptide Receptors. Cells.

[35] Humphries R.K., Dover G., Young N.S., Moore J.G., Charache S., Ley T., Nienhuis A.W. (1985). 5-Azacytidine acts directly on both erythroid precursors and progenitors to increase production of fetal hemoglobin. J. Clin. Investig..

[36] Patil S., Rao R.S., Ganavi B.S. (2015). Mesenchymal-Epithelial Transition in Oral Cancer. J. Int. Oral Health.

[37] Perretti M., Dalli J. (2009). Exploiting the Annexin A1 pathway for the development of novel anti-inflammatory therapeutics. Br. J. Pharmacol..

[38] Cardin L.T., Sonehara N.M., Mimura K.K., Ramos Dinarte Dos Santos A., da Silva WAJunior Sobral L.M., Leopoldino A.M., da Cunha B.R., Tajara E.H., Oliani S.M., Rodrigues-Lisoni F.C. (2017). ANXA1_Ac2-26_ peptide, a possible therapeutic approach in inflammatory ocular diseases. Gene.

[39] Zhang Q., Shi S., Yen Y., Brown J., Ta J.Q., Le A.D. (2010). A subpopulation of CD133(+) cancer stem-like cells characterized in human oral squamous cell carcinoma confer resistance to chemotherapy. Cancer Lett..

[40] Rodini C.O., Lopes N.M., Lara V.S., Mackenzie I.C. (2017). Oral cancer stem cells—Properties and consequences. J. Appl. Oral Sci..

[41] Li J., Li S., Shu M., Hu W. (2024). Unravelling the heterogeneity of oral squamous cell carcinoma by integrative analysis of single-cell and bulk transcriptome data. J. Cell Mol. Med..

[42] Xiao M., Yan M., Zhang J., Xu Q., Qi S., Wang X., Chen W. (2017). Cancer stem-like cell related protein CD166 degrades through E3 ubiquitin ligase CHIP in head and neck cancer. Exp. Cell Res..

[43] Yan M., Yang X., Wang L., Clark D., Zuo H., Ye D., Chen W., Zhang P. (2013). Plasma membrane proteomics of tumor spheres identify CD166 as a novel marker for cancer stem-like cells in head and neck squamous cell carcinoma. Mol. Cell. Proteom..

[44] Ihnen M., Kress K., Kersten J.F., Kilic E., Choschzick M., Zander H., Müller V., Mahner S., Jänicke F., Woelber L. (2012). Relevance of activated leukocyte cell adhesion molecule (ALCAM) in tumor tissue and sera of cervical cancer patients. BMC Cancer.

[45] Hein S., Müller V., Köhler N., Wikman H., Krenkel S., Streichert T., Schweizer M., Riethdorf S., Assmann V., Ihnen M. (2011). Biologic role of activated leukocyte cell adhesion molecule overexpression in breast cancer cell lines and clinical tumor tissue. Breast Cancer Res. Treat..

[46] Lee Y., Shin J.H., Longmire M., Wang H., Kohrt H.E., Chang H.Y., Sunwoo J.B. (2016). CD44+ Cells in Head and Neck Squamous Cell Carcinoma Suppress T-Cell-Mediated Immunity by Selective Constitutive and Inducible Expression of PD-L1. Clin. Cancer Res..

[47] Wang H., Unternaehrer J.J. (2019). Epithelial-mesenchymal Transition and Cancer Stem Cells: At the Crossroads of Differentiation and Dedifferentiation. Dev. Dyn..

[48] Gailhouste L., Liew L.C., Hatada I., Nakagama H., Ochiya T. (2018). Epigenetic reprogramming using 5-azacytidine promotes an anti-cancer response in pancreatic adenocarcinoma cells. Cell Death Dis..

[49] Parker W.B., Thottassery J.V. (2021). 5-Aza-4′-thio-2′-deoxycytidine, a New Orally Bioavailable Nontoxic “Best-in-Class”: DNA Methyltransferase 1-Depleting Agent in Clinical Development. J. Pharmacol. Exp. Ther..

[50] Cihak A., Vesely J., Hynie S. (1980). Transformation and metabolic effects of 5-aza-2′-deoxycytidine in mice. Biochem. Pharmacol..

[51] Thiery J.P., Acloque H., Huang R.Y., Nieto M.A. (2009). Epithelial-mesenchymal transitions in development and disease. Cell.

[52] Oshi M., Tokumaru Y., Mukhopadhyay S., Yan L., Matsuyama R., Endo I., Takabe K. (2021). Annexin A1 Expression Is Associated with Epithelial-Mesenchymal Transition (EMT), Cell Proliferation, Prognosis, and Drug Response in Pancreatic Cancer. Cells.

[53] Rondepierre F., Bouchon B., Papon J., Bonnet-Duquennoy M., Kintossou R., Moins N., Maublant J., Madelmont J.C., D’Incan M., Degoul F. (2009). Proteomic studies of B16 lines: Involvement of annexin A1 in melanoma dissemination. Biochim. Biophys. Acta..

[54] Cheng T.Y., Wu M.S., Lin J.T., Lin M.T., Shun C.T., Huang H.Y., Hua K.T., Kuo M.L. (2012). Annexin A1 is associated with gastric cancer survival and promotes gastric cancer cell invasiveness through the formyl peptide receptor/extracellular signal-regulated kinase/integrin beta-1-binding protein 1 pathway. Cancer.

[55] Maschler S., Gebeshuber C.A., Wiedemann E.M., Alacakaptan M., Schreiber M., Custic I., Beug H. (2010). Annexin A1 attenuates EMT and metastatic potential in breast cancer. EMBO Mol. Med..

[56] Paweletz C.P., Ornstein D.K., Roth M.J., Bichsel V.E., Gillespie J.W., Calvert V.S., Vocke C.D., Hewitt S.M., Duray P.H., Herring J. (2000). Loss of annexin 1 correlates with early onset of tumorigenesis in esophageal and prostate carcinoma. Cancer Res..

[57] Garcia Pedrero J.M., Fernandez M.P., Morgan R.O., Herrero Zapatero A., Gonzalez M.V., Suarez Nieto C., Rodrigo J.P. (2004). Annexin A1 down-regulation in head and neck cancer is associated with epithelial differentiation status. Am. J. Pathol..

[58] Zhang L., Yang X., Zhong L.P., Zhou X.J., Pan H.Y., Wei K.J., Li J., Chen W.T., Zhang Z.Y. (2009). Decreased expression of Annexin A1 correlates with pathologic differentiation grade in oral squamous cell carcinoma. J. Oral Pathol. Med..

[59] Álvarez-Teijeiro S., Menéndez S.T., Villaronga M.Á., Pena-Alonso E., Rodrigo J.P., Morgan R.O., Granda-Díaz R., Salom C., Fernandez M.P., García-Pedrero J.M. (2017). Annexin A1 down-regulation in head and neck squamous cell carcinoma is mediated via transcriptional control with direct involvement of miR-196a/b. Sci. Rep..

[60] Tian C., Chen K., Gong W., Yoshimura T., Huang J., Wang J.M. (2020). The G-Protein Coupled Formyl Peptide Receptors and Their Role in the Progression of Digestive Tract Cancer. Technol. Cancer Res. Treat..

[61] Vacchelli E., Ma Y., Baracco E.E., Sistigu A., Enot D.P., Pietrocola F., Yang H., Adjemian S., Chaba K., Semeraro M. (2015). Chemotherapy-induced antitumor immunity requires formyl peptide receptor 1. Science.

[62] Gastardelo T.S., Cunha B.R., Raposo L.S., Maniglia J.V., Cury P.M., Lisoni F.C., Tajara E.H., Oliani S.M. (2014). Inflammation and cancer: Role of annexin A1 and FPR2/ALX in proliferation and metastasis in human laryngeal squamous cell carcinoma. PLoS ONE.

[63] Flavell R.A., Sanjabi S., Wrzesinski S.H., Licona-Limón P. (2010). The polarization of immune cells in the tumour environment by TGFbeta. Nat. Rev. Immunol..

[64] Sautès-Fridman C., Cherfils-Vicini J., Damotte D., Fisson S., Fridman W.H., Cremer I., Dieu-Nosjean M.C. (2011). Tumor microenvironment is multifaceted. Cancer Metastasis Rev..

