# Supplementary material for: Annexin A1 Is Involved in the Antitumor Effects of 5-Azacytidine in Human Oral Squamous Carcinoma Cells
Source: Cancers (Basel). 2025 Mar 21;17(7):1058. doi: 10.3390/cancers17071058 (PMC11988024; doi:10.3390/cancers17071058)
Supplement: Supplementary file 1 [file cancers-17-01058-s001.zip › Supplementary Figure S1.pdf]

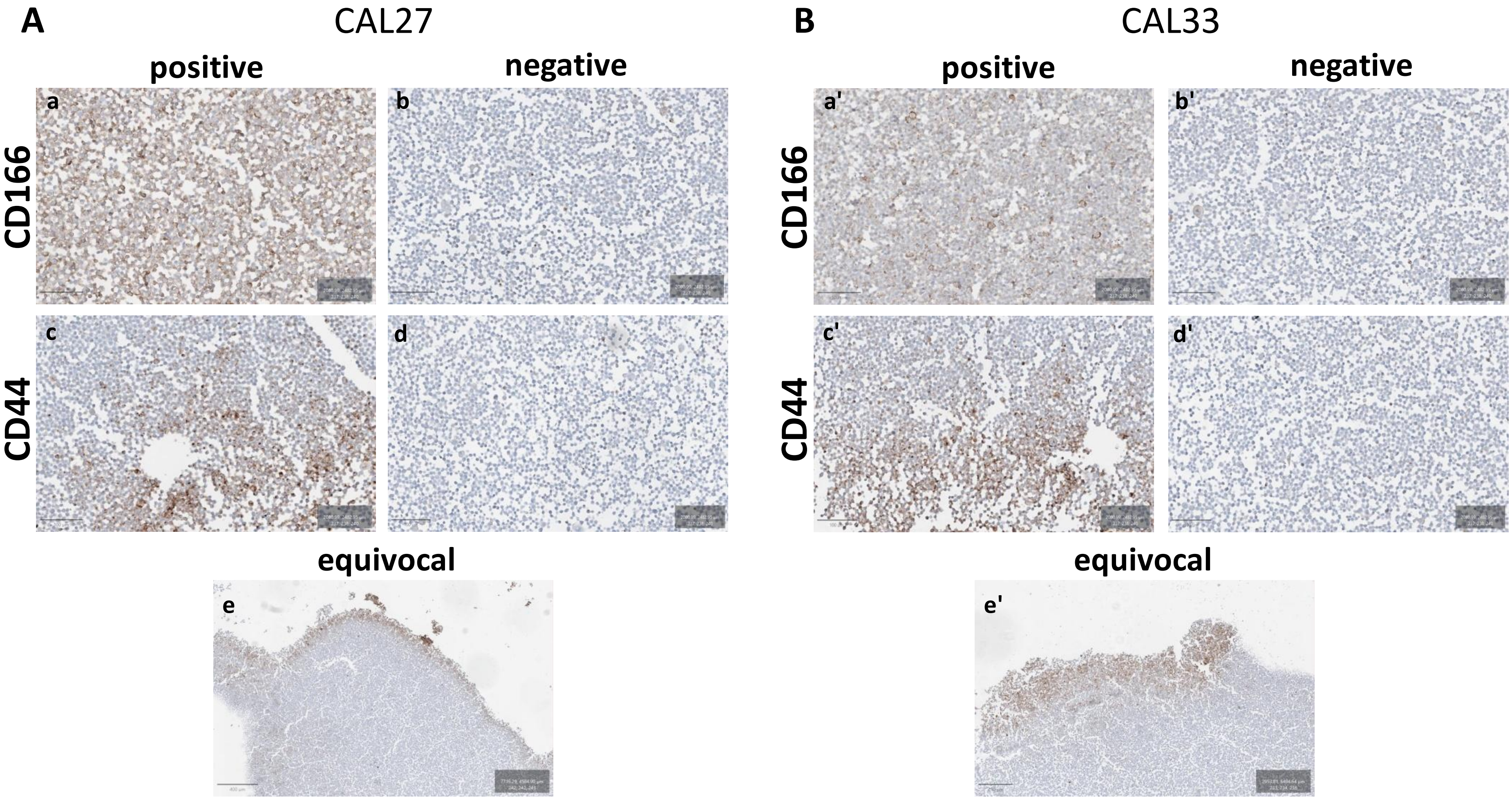

Figure S1: Representative images for IHC quantification of CD44 and CD166 on CAL27 (A) and CAL33 (B) cells. Positive: demonstrating the target marker's distinct and readily appreciable presence, with a clear staining pattern in ≥10% of tumor cells. Positivity for CD166 and CD44 was visualized as brown membrane and/or cytoplasmic staining (a, a’ and c , c’, respectively); negative: displaying the absence of specific staining or staining considered artifactual, or <=10% of tumor cells showing positive staining (b, b’ and d, d’); equivocal: showing a weak, ambiguous, or focal staining that did not meet the criteria for positive or negative (e, e’).Two pathologists independently scored the immunostaining level of these antibodies semiquantitatively [S.S. and G.I.]. In cases of discrepancy between the two pathologists, a third independent pathologist was consulted to reach a final consensus score.
