# Supplementary material for: Annexin A1 Is Involved in the Antitumor Effects of 5-Azacytidine in Human Oral Squamous Carcinoma Cells
Source: Cancers (Basel). 2025 Mar 21;17(7):1058. doi: 10.3390/cancers17071058 (PMC11988024; doi:10.3390/cancers17071058)
Supplement: Supplementary file 1 [file cancers-17-01058-s001.zip › Supplementary Figure S2.pdf]

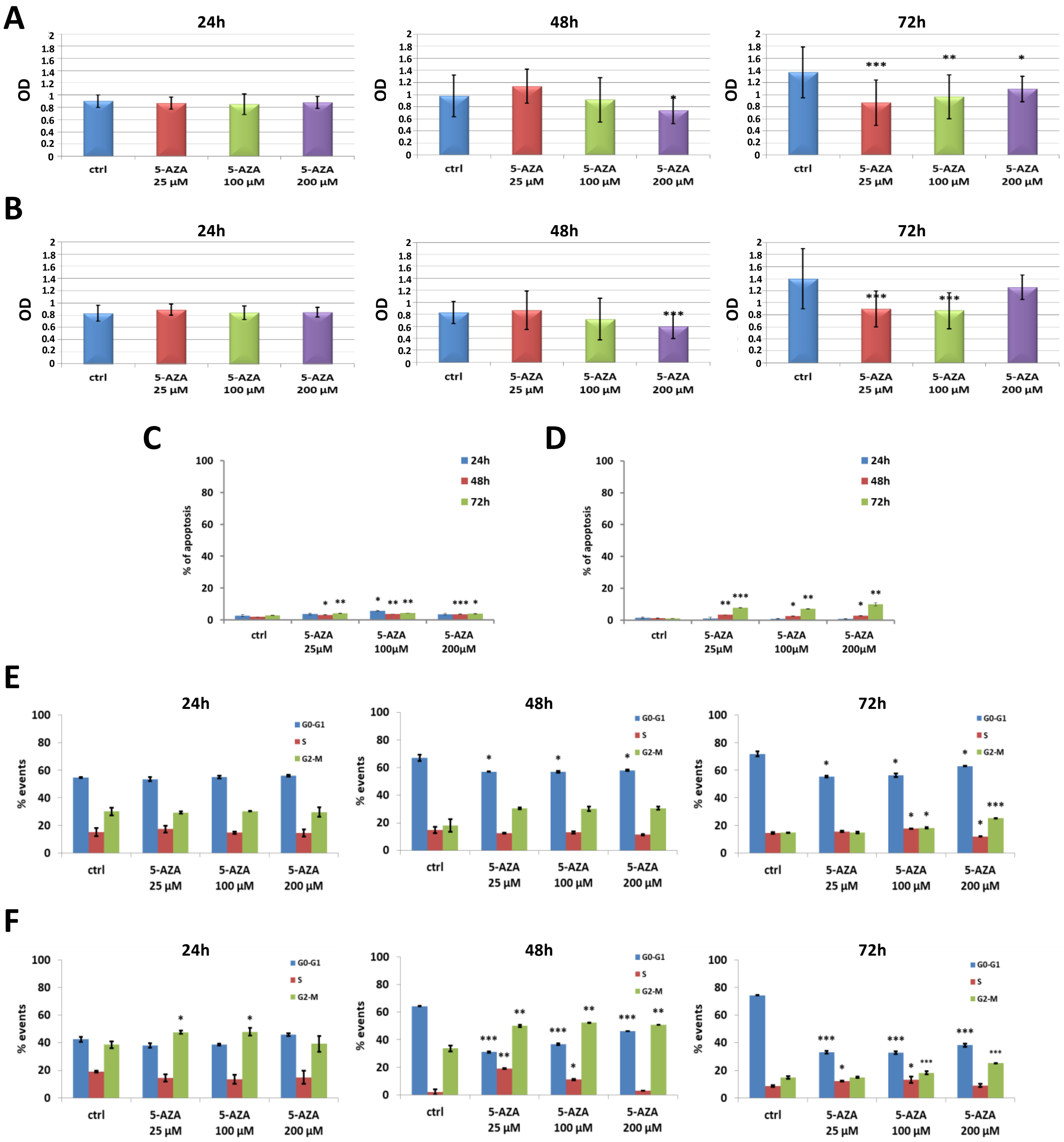


**Figure S1**: Analysis of 5-AZA effects on cell death and cycle of CAL27 and CAL33 cells. MTT assay performed on CAL27 (A) and CAL33 (B) with 5-AZA at concentration of 25 µM, 100 µM and 200 µM at 24 h, 48 h, 72 h. Apoptosis of CAL27 (C) and CAL33 (D), and cell cycle analysis of CAL27 (E) and CAL33 (F) after 5-AZA treatment at concentration of 25 µM, 100 µM and 200 µM for 24 h, 48 h and 72 h. Data represent the mean of three independent experiments ± S.D. with similar results; error bars represented the S.D. $*p < 0.05$, $**p < 0.01$, $***p < 0.001$ *versus* untreated control.
