# Supplementary material for: Annexin A1 Is Involved in the Antitumor Effects of 5-Azacytidine in Human Oral Squamous Carcinoma Cells
Source: Cancers (Basel). 2025 Mar 21;17(7):1058. doi: 10.3390/cancers17071058 (PMC11988024; doi:10.3390/cancers17071058)
Supplement: Supplementary file 1 [file cancers-17-01058-s001.zip › Supplementary Figure S3 .pdf]

A

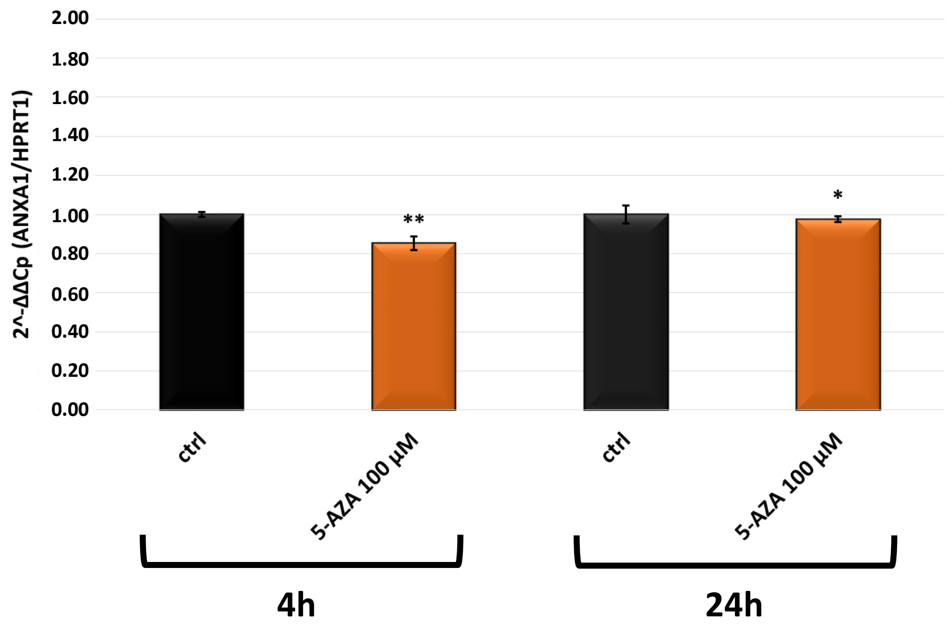

Figure S3: Analysis of ANXA1 mRNA expression in CAL27. (A) ANXA1 mRNA levels were detected in CAL27 cells after treatment with 5-AZA at concentration of 100 µM for 4 and 24 hr by Real Time- Polymerase Chain Reaction (RT-PCR) . The mRNA ANXA1 expression was performed on levels of HPRT1. Values obtained were expressed using the delta-delta Ct method to derive relative fold change. Data represent the mean of three independent experiments ± S.D. with similar results; error bars represented the S.D. *$p < 0.05$, **$p < 0.01$ *versus* untreated corresponding control.
